# Association between physical activity and chronic disease multimorbidity patterns in Chinese middle-aged and older adults

**DOI:** 10.3389/fmed.2025.1582846

**Published:** 2025-09-29

**Authors:** Xiaodai Wen, Xinmei Yang

**Affiliations:** School of Public Health and Health Management, Fujian Health College, Fuzhou, China

**Keywords:** physical activity, chronic disease, multimorbidity pattern, middle-aged and older adults, China

## Abstract

**Background:**

The growing burden of chronic disease multimorbidity in an aging population highlights the need to promote physical activity as a key strategy for disease management. This study aimed to explore the patterns of chronic comorbidities and their association with physical activity in Chinese middle-aged and older adults.

**Methods:**

A cross-sectional study was conducted using data from the 2020 China Health and Retirement Longitudinal Study. Latent class analysis was applied to identify distinct multimorbidity patterns among middle-aged and older adults, whereas multivariate logistic regression was used to analyze the influencing factors. The χ^2^ test was performed to compare 10 categorical variables between the patterns. A total of 18,697 participants were included.

**Results:**

Chronic disease multimorbidities were categorized into three classes: Class 1 (Metabolic Pattern), Class 2 (Multisystem Pattern), and Class 3 (Hypertension-Digestive-Musculoskeletal Pattern). Engagement in moderate-intensity physical activity was associated with a lower odds of Multisystem Pattern (OR = 0.74, 95% CI: 0.70∼0.91). Engagement in high-intensity physical activity was linked to a lower odds of both Metabolic Pattern (OR = 0.75, 95% CI: 0.68∼0.83) and Multisystem Pattern (OR = 0.75, 95% CI: 0.67∼0.84) diseases but was associated with a higher odds of Hypertension-Digestive-Musculoskeletal Pattern (OR = 1.46, 95% CI: 1.34∼1.59) diseases.

**Conclusion:**

Moderate physical activity reduces the risk of Multisystem Pattern and plays an essential role in preventing and managing metabolic disorders. Although high-intensity physical activity can reduce the risk of metabolic disorders and Multisystem Pattern, excessive physical activity may increase the risk of Hypertension-Digestive-Musculoskeletal Pattern.

## 1 Introduction

As the global population ages, the prevalence of chronic diseases is increasing, and as is the co-occurrence of multiple long-term conditions (1). This can lead to reduced quality of life for patients (2), increased utilization of inpatient and outpatient services (3), and increased complexity for clinical care and patient management strategies (4). The situation is particularly true in low- and middle-income countries (5). The current research mostly focus on how exercise can help prevent single diseases (6). It is no longer to address the current situation of In response, China recently launched a plan to prevent and control chronic diseases, aiming to restructure the health service system to better manage multimorbiditiy chronic diseases (7). Exploring the prevalence trends and factors related to chronic diseases in middle and older adults, especially the influence of low-cost physical activity (PA) on chronic diseases, can help to improve strategies for the prevention and control of chronic diseases.

The World Health Organization guidelines strongly recommend that older adults engage in 150–300 min of moderate-intensity PA/week or 75–150 min of high-intensity PA or an equivalent combination (8). PA not only effectively prevents cardiovascular diseases, metabolic diseases and respiratory diseases, but also improves the health conditions of patients suffering from these chronic diseases (9). Therefore, reaching the recommended levels of PA is a key in strategies for managing chronic diseases. The previous study was conducted in high-income countries, with findings ranging from an inverse correlation to no effect between PA and multimorbidity (10). However, the patterns of PA, disease prevalence rates and healthcare systems are quite different from other countries (9), especially in the context aging population and the increasing burden of chronic diseases (11). Therefore, exploring the association between multimorbidity patterns in the middle-aged and older adults in China is necessary to provide more precise guidance for effective interventions (10).

In this study, data from the China Health and Retirement Longitudinal Study (CHARLS) were used to examine the current state of chronic multimorbidity in middle-aged and older Chinese adults. Our main aim was to understand the impact of varying PA levels on multimorbidity patterns within this population. The findings would provide a theoretical basis for chronic disease prevention and offer guidance for the development of related health policies and optimization of intervention measures.

## 2 Materials and methods

### 2.1 Data sources

We used data from the CHARLS, a large interdisciplinary survey conducted by Wuhan and Peking universities. The national baseline survey of CHARLS was conducted in 2011, and since then, national follow-up surveys have continued in 2013, 2015, 2018, and 2020, covering 450 villages and 28 provinces in China (11).

Detailed variable definitions and coding schemes used in this study were based on the CHARLS 2020 dataset. The supplementary codebook (see Supplementary material) provides an overview of the variable labels, coding strategies, and construction of physical activity measures according to the IPAQ guidelines.

The data used in this study were from the fifth round of surveys that took place in 2020. The questions including information on the living habits and chronic diseases of middle-aged and older adults in this survey. The inclusion criteria for participants were: (1) age of ≥ 45 years at the time of the baseline survey; (2) available data on smoking, alcohol consumption, education level, cohabitation status, self-reported sleep duration, level of PA, and physician-diagnosed chronic diseases. After screening, 18,697 participants were included.

### 2.2 Data collection

#### 2.2.1 Chronic disease and multimorbidity measurements

The demographic background, health status, and functioning data were taken from the CHARLS 2020 questionnaire. The prevalence of chronic diseases could be identified by asking, “Have you been diagnosed by a doctor with [the following diseases]?” to assess the presence of diseases including hypertension; dyslipidemia (elevated low-density lipoprotein, triglycerides, and total cholesterol, or decreased high-density lipoprotein); diabetes or hyperglycemia; cancer or malignant tumors (excluding minor skin cancer); chronic lung diseases (such as chronic bronchitis or emphysema, excluding tumors or cancers); liver diseases (excluding fatty liver, tumors and cancers); heart disease (including coronary heart disease, angina pectoris, congestive heart failure or other heart diseases); stroke; kidney diseases (excluding tumors or cancers); diseases of the stomach or other digestive systems (excluding tumors or cancers); emotional, neurological, or mental problems; memory-related diseases (such as dementia, brain atrophy, Parkinson’s disease, etc.); arthritis or rheumatism, and asthma. A total of 14 common chronic diseases were included.

#### 2.2.2 Physical activity measurements

The physical activity section of the questionnaire directly used the short version of the International Physical Activity Questionnaire (IPAQ) (12), a global questionnaire designed to measure total PA across all areas. According to the questionnaire, the daily PA time of the respondents was divided into five groups: 0, 10–29, 30–119, 120–239 min, and > 4 h. The duration of each intensity level was replaced by the median of each group (the > 4 h group was replaced by 4 h) (13). Metabolic equivalent (MET), an indicator of the intensity of PA, was used to calculate the amount of moderate to vigorous PA (14). Based on the IPAQ criteria (15), the MET is 3.3 for walking, for moderate-intensity PA is 4.0, and for high-intensity PA is 8.0. The PA was calculated as follows:

Total PA (METs/week) = 8.0 × total weekly duration of high-intensity PA + 4.0 × total weekly duration of moderate PA + 3.3 × total weekly duration of walking

According to the IPAQ, weekly levels of PA were categorized as low-intensity < 600 (METs/week), moderate-intensity 600–3,000 (METs/week), and high-intensity > 3,000 (METs/week) (16).

#### 2.2.3 Covariates

In previous studies the effects associated with kidney disease and sarcopenia include personal factors such as gender, age and comorbidities, and also socioeconomic factors and living standards (17–19). Meta-analyses indicate that smoking is an independent risk factor for the development of chronic diseases (20) and sarcopenia (21). The effects of alcohol on the kidney in comparison to smoking remain controversial in the scientific community (22). Study has shown that the relationship between sleep and chronic diseases is not a simple linear one (23). The duration of sleep at night shows a U-shaped relationship with the risk of cardiovascular or cerebrovascular diseases. Those who sleep for approximately 7.5 h each night have the lowest risk of developing such diseases (24). Therefore, our research collected information on sociodemographic conditions and health-related factors. These factors included age, sex, education level, spouse status, smoking, alcohol drinking, and sleep duration.

Based on existing evidence (25), sleep duration of < 7 h is associated with cardiovascular diseases, diabetes, and poor self-rated health. Therefore, sleep duration was categorized as < 7 or ≥ 7 h. Educational level was categorized into high school and above and below high school based on China’s compulsory education policy.

### 2.3 Statistical analysis

#### 2.3.1 Comparison of covariates across multimorbidity patterns

The χ^2^ test was used to compare the distribution of covariates across the three chronic disease multimorbidity patterns.

#### 2.3.2 Regression analysis of physical activity and multimorbidity

Separate binary logistic regression models were conducted for each latent class to examine the association between physical activity and multimorbidity patterns identified through latent class analysis (LCA). In each model, the outcome variable was coded as 1 if the individual belonged to the specified class and 0 if not. Level of physical activity (moderate intensity and high intensity) was used as the main exposure variable, and the types of chronic diseases (Class 1, Class 2, Class 3) were the outcome variables. The regression model controlled for multiple potential confounding factors, including age, gender, history of smoking and drinking, sleep duration, educational level, and whether living with a spouse.

#### 2.3.3 Latent class analysis

Latent class analysis (LCA) is a cross-sectional latent variable mixture modeling approach (26). It does this by analyzing the behavior patterns of individuals, and finding common types called classes (27). The LCA model calculates the conditional probability of each disease within each latent class based on the presence or absence of chronic diseases among participants, thereby identifying distinct multimorbidity patterns. This leads to individual subgroups where they are most similar to each other and most different from those in other classes (28). The key advantage of model-based techniques over heuristic clustering techniques (e.g., K-means) is that they provide appropriate statistical data (29). Model selection was based on commonly used LCA fit statistics, including Akaike information criteria (AIC), Bayesian information criteria (BIC), and sample size-adjusted BIC (saBIC) (30). These criteria are used to compare competing models, with lower values indicating better model fit. Among them, BIC is often preferred for its stricter penalty on model complexity, especially in larger samples. Lower AIC and BIC values indicate better model fit. Classification accuracy was evaluated using entropy, which ranges from 0 to 1. The closer the entropy is to 1, the more accurate the classification is. When the entropy index is 0.6, the classification accuracy exceeds 80% (31). Lastly, LMR and BLRT were used to evaluate the fitting differences of the LCA model. These tests evaluate fitting differences, whether the k-class model fits significantly better than the k–1 class model (32). Fit statistics help researchers select the most suitable model for the data, and can be used to compare models for hypothesis testing. Therefore, LCA is an effective tool for understanding the multiple chronic disease conditions of middle-aged and elderly people, and identifying multimorbidity patterns. Supplementary Table 2 provides the definitions and functions of the LCA model fit indices.

Latent class analysis of comorbidity chronic diseases in middle-aged and older adults was conducted using Mplus software (version 8.3). SPSS (version 22.0) was used to analyze the factors that influenced decisions and compare the groups in each category. All statistical tests were two-sided with a significance level of α = 0.05.

## 3 Results

### 3.1 Participants characteristics

Of the 18,697 participants, 47.62% were male and 52.38% were female. Overall, 37.84% of participants were aged ≥ 65 years, and 62.16% were aged < 65 years. The proportion of participants who drank alcohol was 36.22%, and the proportion of those who did not was 63.78%. Smokers accounted for 39.22%, and non-smokers accounted for 60.78%.

In terms of sleep duration, 60.11% of participants slept for less than 7 h each night, and 39.89% slept for 7 h or more. With respect to educational attainment, 13.07% had a high school education or above, and 86.93% had a junior high school education or below. In terms of living conditions, 84.10% of the residents lived with their spouse, and 15.90% did not (Table 1).

**TABLE 1:** Characteristics of the participants.

Variables	Total participants	Low-intensity PA	Moderate-intensity PA	High-intensity PA
	(*N* = 18,697)	(*N* = 3,151)	(*N* = 4,987)	(*N* = 10,559)
**Gender, *N* (%)**				
Male	8,904 (47.62)	1,433 (45.47)	2,399 (48.11)	5,072 (48.07)
Female	9,793 (52.38)	1,718 (54.53)	2,588 (51.89)	5,487 (51.93)
**Age, *N* (%)**				
≥ 65	7,076 (37.84)	1,679 (53.28)	2,069 (41.48)	3,328 (31.52)
45 ≤ and < 65	11,621 (62.16)	1,472 (46.72)	2,918 (58.52)	7,231 (68.48)
**Drinking, *N* (%)**				
Yes	6,772 (36.22)	896 (28.43)	1,758 (35.25)	4,118 (39.00)
No	11,925 (63.78)	2,255 (71.57)	3,229 (64.75)	6,441 (61.00)
**Smoking, *N* (%)**				
Yes	7,333 (39.22)	1,264 (40.11)	1,964 (39.38)	4,105 (38.88)
No	1,1364 (60.78)	1,887 (59.89)	3,023 (60.62)	6,454 (61.12)
**Sleep time, *N* (%)**				
≥ 7 h	7,459 (39.89)	1,299 (41.22)	1,961 (39.32)	4,199 (39.77)
< 7 h	11,238 (60.11)	1,852 (58.77)	3,026 (60.68)	6,360 (60.23)
**Education level, *N* (%)**				
High school and above	2,445 (13.07)	282 (8.95)	937 (18.79)	1,226 (11.61)
Below high school	16,252 (86.93)	2,869 (91.05)	4,050 (81.21)	9,333 (88.39)
**Living with spouse, *N* (%)**				
With a spouse	15,725 (84.10)	2,373 (75.31)	4,121 (82.63)	9,231 (87.42)
Without a spouse	2,972 (15.90)	778 (24.69)	866 (17.37)	1,328 (12.58)

**FIGURE 1:**
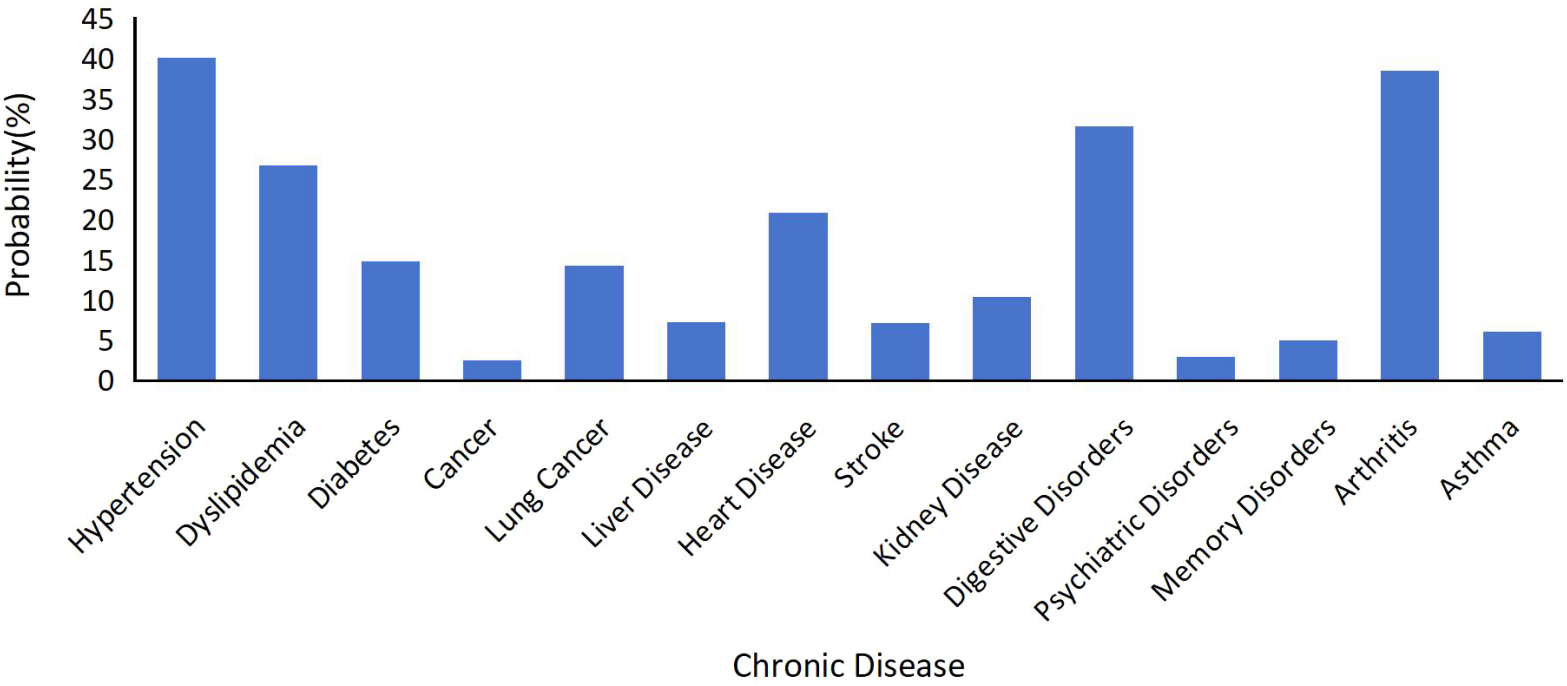
Descriptive of chronic disease probability.

The prevalence rates of various chronic diseases were hypertension 40.12%, arthritis 38.55%, digestive system diseases 31.57%, dyslipidemia 26.74%, and heart disease 20.89% (Figure 1).

### 3.2 Classification of chronic disease multimorbidity patterns in middle-aged and older Chinese adults

Fitting index data are shown in Table 2. Entropy values of the latent category model were 0.641 for two categories, 0.681 for three categories, 0.632 for four categories, 0.656 for five categories, and 0.633 for six categories, and in LMR and BLRT the significance of this observation was *p* < 0.001. Chronic disease multimorbidities were divided into three classes based on 14 chronic diseases. Thus, models with three categories were selected as the best model due to high entropy values and better-represented groups of chronic disease multimorbidity.

**TABLE 2:** Fit indices of latent class analysis and the distribution rates of chronic disease multimorbidity patterns.

Cluster	AIC	BIC	SaBIC	Entropy	LMRT (P)	BLRT (P)	Latent class distribution rate (%)					
							1	2	3	4	5	6
2-class	192933.571	193160.819	193068.658	0.641	*P* < 0.001	*P* < 0.001	0.300	0.700				
3-class	190759.729	191104.518	190964.688	0.681	*P* < 0.001	*P* < 0.001	0.217	0.130	0.652			
4-class	189767.060	190229.398	190041.899	0.632	*P* < 0.001	*P* < 0.001	0.053	0.232	0.137	0.578		
5-class	188896.007	189475.880	189240.71	0.656	*P* < 0.001	*P* < 0.001	0.036	0.052	0.228	0.526	0.158	
6-class	188728.141	189425.556	189142.719	0.633	0.116	*P* < 0.001	0.168	0.055	0.055	0.033	0.176	0.514

Class 1 had 4,066 participants, with a high prevalence of hypertension, dyslipidemia, and diabetes; thus, class 1 was named “Metabolic Pattern.” Class 2 had 2,433 participants, with a high prevalence of hypertension, dyslipidemia, diabetes, arthritis, and lung, liver, heart, kidney, digestive, and psychiatric diseases. Class 2 involved several systems and was defined as a “Multisystem Pattern.” Class 3 contained 12,198 samples, with a high prevalence of hypertension, digestive diseases, and arthritis, and it was defined as a “Hypertension-Digestive-Musculoskeletal Pattern” (33).

**FIGURE 2:**
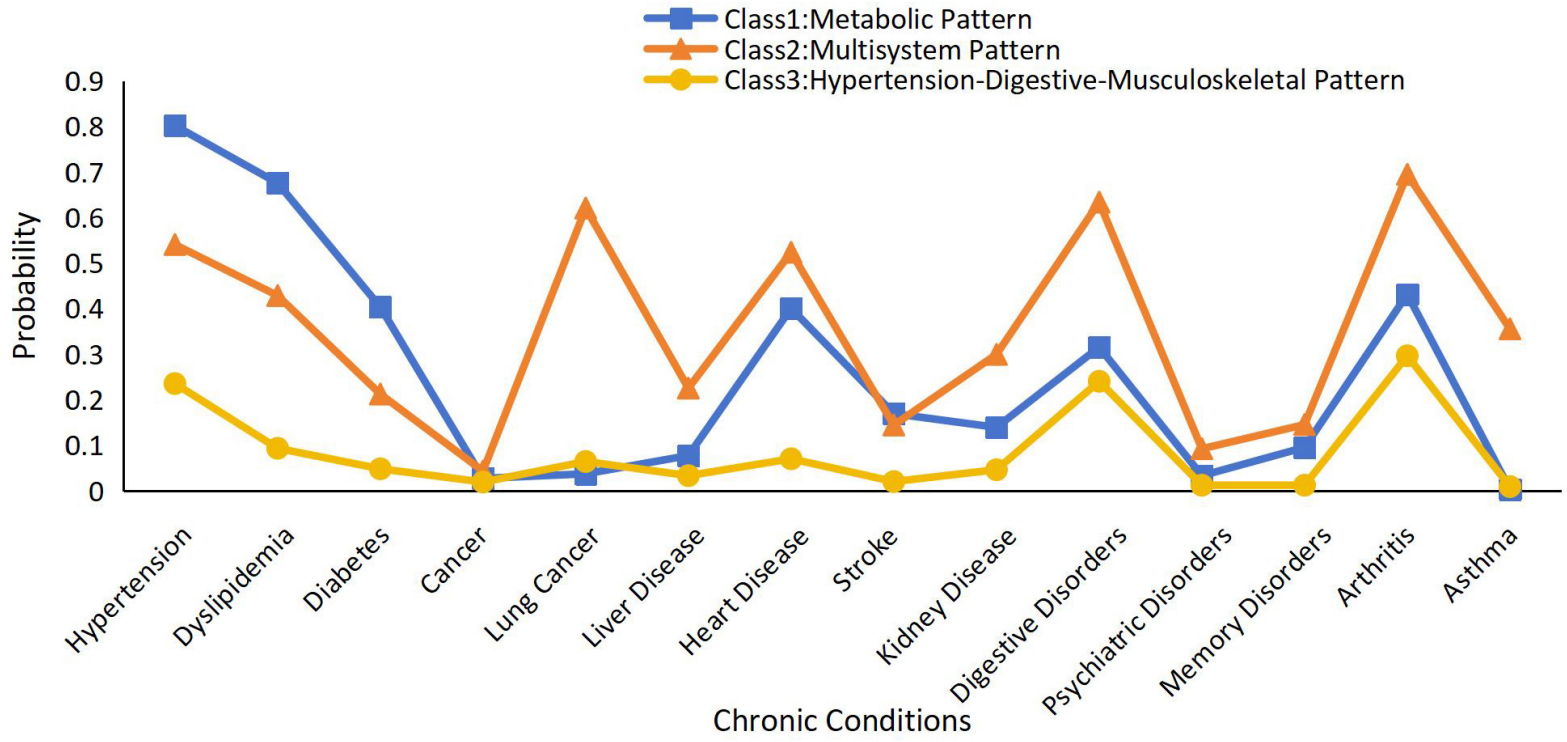
Patterns of chronic multimorbidity by latent classes analysis.

The conditional probability distributions for each class are shown in Figure 2. Metabolic Pattern was characterized by a high probability of hypertension, dyslipidemia, and diabetes, but low probabilities for other conditions. Multisystem Pattern showed moderate-to-high probabilities across a wide range of conditions, including cancer, heart disease, digestive disorders, arthritis, and psychiatric disorders, indicating a complex multimorbidity profile. Hypertension-Digestive-Musculoskeletal Pattern had the highest proportion of participants and was mainly marked by elevated probabilities of hypertension, digestive disorders, and arthritis.

The results of the latent class analysis are summarized in Supplementary Table 1, which presents the conditional item-response probabilities of reporting each chronic condition within the three identified classes.

### 3.3 Comparison of demographic and PA characteristics across different categories

Participants engaging in moderate-intensity PA had the highest proportion of Class 1 multimorbidities (31.65%). Participants engaging in high-intensity PA had the highest proportion of Class 3 multimorbidities (60.19%).

Sociodemographic and health-related factors varied across the classes. Class 1 had the highest proportion of females (55.51%), and it also had the highest proportion of participants with an educational level ≥ high school (15.69%). Class 2 had the highest proportion of participants aged ≥ 65 years (52.60%), and it also had the highest proportion of participants with a history of smoking (43.00%). Class 3 had the highest proportion of participants with a history of alcohol consumption (39.00%). Participants living with spouses had the highest proportion of multimorbidities in Class 3 (85.67%) (Table 3).

**TABLE 3:** Comparison of characteristics among latent classes in middle-aged and older Chinese adults (*n*, %).

Variables		Class 1 (n = 4,066)	Class 2 (n = 2,433)	Class 3 (n = 12,198)	χ2	P
**Personal characteristics**						
Sex	Male	1,809 (44.49)	1,139 (46.81)	5,956 (48.82)	23.73	0 < 0.001
	Female	2,257 (55.51)	1,294 (53.19)	6,242 (51.18)		
Age (years)	≥ 65	1,862 (45.79)	1,280 (52.60)	3,934 (32.25)	496.98	0 < 0.001
	< 65 and ≥ 45	2,204 (54.21)	1,153 (47.40)	8,264 (67.75)		
Drink	Yes	1,298 (31.92)	713 (29.30)	4,761 (39.00)	124.57	0 < 0.001
	No	2,768 (68.08)	1,720 (70.70)	7,437 (61.00)		
Smoke	Yes	1,444 (35.51)	1,045 (43.00)	4,844 (39.71)	38.87	0 < 0.001
	No	2,622 (64.49)	1,388 (57.00)	7,354 (60.29)		
Sleep time (h)	≥ 7	1,484 (36.49)	688 (28.27)	5,287 (43.34)	216.99	0 < 0.001
	< 7	2,582 (63.51)	1,745 (71.73)	6,911 (56.66)		
Education level	High school and above	649 (15.96)	243 (10.00)	1,553 (12.73)	51.47	0 < 0.001
	Below high school	3,417 (84.04)	2,190 (90.00)	10,645 (87.27)		
Living with spouse	Yes	3,368 (82.83)	1907 (78.38)	10,450 (85.67)	86.90	0 < 0.001
	No	698 (17.17)	526 (21.62)	1,748 (14.33)		
Low-intensity PA	Yes	791 (19.45)	549 (22.56)	1,811 (14.85)	111.31	0 < 0.001
	No	3,275 (80.55)	1,884 (77.44)	10,387 (85.15)		
Moderate-intensity PA	Yes	1,287 (31.65)	654 (26.88)	3,046 (25.00)	69.67	0 < 0.001
	No	2,779 (68.35)	1,779 (73.12)	9,152 (75.00)		
High-intensity PA	Yes	1,988 (48.89)	1,230 (50.55)	7,341 (60.18)	197.97	0 < 0.001
	No	2,078 (51.11)	1,203 (49.45)	4,857 (39.82)		

Class 1: Metabolic Pattern. Class 2: Multisystem Pattern. Class 3: Hypertension-Digestive-Musculoskeletal Pattern.

### 3.4 Logistic regression analysis of potential factors of chronic disease multimorbidity patterns

When investigating associations between participant characteristics and multimorbidity classes, distinct patterns emerged across classes (Table 4). In Class 1, aged ≥ 65 years was associated with increased odds of classification (OR = 1.53, 95% CI: 1.42∼1.65), as was living with a spouse (OR = 1.49, 95% CI: 1.35∼1.65). High-intensity PA was associated with decreased odds of classification (OR = 0.75, 95% CI: 0.68∼0.83). In Class 2, aged ≥ 65 years was associated with increased odds of classification (OR = 1.77, 95% CI: 1.62∼1.94), as was sleep duration of ≥ 7 h (OR = 1.47, 95%CI: 1.29∼1.67). Both moderate-intensity PA (OR = 0.74, 95% CI: 0.70∼0.90) and high-intensity PA (OR = 0.75, 95% CI: 0.60∼0.84) were associated with decreased odds of Class 2. In Class 3, history of smoking (OR = 1.35, 95% CI: 1.26∼1.45) and educational level of high school or above (OR = 1.53, 95% CI: 1.43∼1.63) were associated with increased odds of this pattern. High-intensity PA was associated with increased odds of classification Class 3 (OR = 1.46, 95% CI: 1.34∼1.59).

**TABLE 4:** Logistic regression models of latent class analysis of chronic disease multimorbidity patterns.

Variables	Class 1		Class 2		Class 3	
	OR	95% CI	OR	95% CI	OR	95% CI
Sex (ref. = female)	0.97	0.87∼1.07	0.91	0.79∼1.03	1.08	0.98∼1.18
Age (ref. = < 65 years)	1.53**	1.42∼1.65	1.77**	1.62∼1.94	0.54**	0.50∼0.58
Drink (ref. = no)	1.10	0.99∼1.21	0.85*	0.76∼0.95	1.03	0.94∼1.12
Smoke (ref. = no)	0.85**	0.79∼0.93	0.70**	0.63∼0.77	1.35**	1.26∼1.45
Sleep time (ref. = < 7 h)	0.86*	0.77∼0.95	1.47**	1.29∼1.67	0.93	0.85∼1.01
Education level (ref. = below high school)	0.84**	0.78∼0.90	0.55**	0.50∼0.61	1.53**	1.43∼1.63
Living with spouse (ref. = no)	1.49**	1.35∼1.65	0.85*	0.74∼0.99	0.78**	0.71∼0.85
Moderate–intensity PA (ref. = no)	1.05	0.95∼1.17	0.74**	0.70∼0.90	1.09	1.00∼1.20
High–intensity PA (ref. = no)	0.75**	0.68∼0.83	0.75**	0.67∼0.84	1.46**	1.34∼1.59

**P < 0.001;

*P < 0.01; OR, odds ratio; 95% CI, 95% confidence interval.

## 4 Discussion

### 4.1 Effect of PA on chronic disease multimorbidity patterns

This study revealed that individuals who engaged more frequently in moderate-intensity PA were less likely to exhibit a multimorbidity pattern. Previous studies have reported that moderate-intensity PA can effectively reduce systemic inflammation levels and improve metabolic indicator (34–36), furthermore, such PA can significantly control the risk of all-cause mortality, especially among middle-aged and elderly people (37).

Participants who engaged in high-intensity PA were less likely to exhibit the Metabolic Pattern or the Multisystem Pattern (Class 3). However, participants who engaged in high-intensity PA were more likely to exhibit the Hypertension-Digestive-Musculoskeletal Pattern. A previous study revealed that regular high-intensity PA can reduce the risks of cancer, obesity and all-cause mortality and improve the overall health status of elderly individuals (38). However, some studies have reported that high-intensity PA can increase cardiovascular load among elderly individuals (39), which in turn can lead to abnormal arterial function (40). In addition, during high-intensity PA, the musculoskeletal load increases significantly. As elderly individuals have a degenerated skeletal system, they may thus be more susceptible to the induction or aggravation of bone diseases (41). Therefore, the recommendation of high-intensity PA should be based on a scientific assessment of individual health conditions, especially among middle-aged and elderly individuals. The functional status of their cardiovascular and skeletal systems should be considered with the aims of minimizing exercise-related risks and maximizing their health-promoting benefits.

In conclusion, PA plays a significant role in the prevention and control of chronic diseases among middle-aged and elderly people; however, an appropriate intensity of activity should be selected on the basis of individual health conditions. Moderate-intensity PA is more suitable for most elderly people and can be used as a routine intervention method. Although high-intensity PA offers certain benefits, it should be performed under professional guidance and monitoring, especially among individuals with cardiovascular or skeletal diseases, and caution should be exercised in this context.

### 4.2 Effects of participant’s characteristics and lifestyles on chronic disease multimorbidity patterns

In the current study, participants aged ≥ 65 years exhibited significantly lower odds of inclusion in the hypertension-digestive-musculoskeletal pattern group, thus suggesting that such chronic conditions may be more common among individuals between the ages of 45 and 65 years. This finding is consistent with the extant research on hypertension and musculoskeletal disease (42). Previous studies have reported that hormonal fluctuations in women between the ages of 45 and 60 years can significantly affect both systolic and diastolic blood pressure; furthermore, these effects are more notable among individuals who experience the onset of menopause earlier (43, 44). Therefore, more attention should be given to blood pressure monitoring among individuals in this age group.

Smoking was a significant variable among individuals in the hypertension-digestive-musculoskeletal pattern group in our study. This finding is consistent with the results reported by previous studies that have indicated that smoking is a risk factor for chronic diseases such as hypertension and arthritis (45). Harmful substances such as the nicotine in tobacco can increase the risk of cardiovascular diseases (46) and related multimorbidities by damaging the vascular endothelium and promoting atherosclerosis (47).

Participants who had obtained higher levels of education were more likely to be classified as part of the hypertension-digestive-musculoskeletal pattern group. This finding is consistent with the results of some studies that have been conducted in developing countries (48), thus suggesting that relationships between level of education and chronic diseases may vary depending on the individual’s socioeconomic background or the stage of epidemiological transition, and these relationships may involve complex interactions among factors such as health behaviors, resource acquisition paths, and the ability to transform health knowledge (49).

Compared with individuals who lived alone, participants who lived with a spouse were less likely to be assigned to the hypertension-digestive-musculoskeletal pattern group, which is consistent with the findings of previous research on spousal care in the United States (50). The reason for this situation could be that a spouse can provide humanistic care and support efforts to maintain a healthy diet, thereby contributing to improved control of blood pressure, blood glucose, and other health metrics among older adults with chronic diseases (51).

The results of the present study indicate that PA recommendations should be formulated for people of different ages and with different educational backgrounds to delay the progression of comorbidities.

### 4.3 Limitations and strengths

First, although the CHARLS 2020 data provided sampling weights, we did not adopt weighted processing in our analysis. This study is a cross-sectional one, with the main aim of evaluating the internal association between physical activity and the comorbidity patterns of chronic diseases. Existing methodological studies have suggested that if the model already includes key sampling design variables (such as age, sex, and region), the unweighted regression model can still obtain effective inferences while avoiding the increase in estimated variance caused by weighting.

Second, although this study controlled for multiple sociodemographic and behavioral variables based on previous research, it did not use the directed acyclic graph (DAG) method to systematically identify potential confounding factors. Therefore, some important but unobserved variables (such as stress, depression, social support, and other psychosocial factors) were not included in the analysis model (52). This may affect the completeness and explanatory power of the results (53, 54).

Finally, this study utilized the cross-sectional data from CHARLS 2020. As a result, it was impossible to infer a causal relationship between physical activity and comorbidities of chronic diseases. Longitudinal studies are still needed in the future to further verify the directionality and sustainability of this association.

This study had several strengths. First, it used the LCA method, which, can identify potential sub-populations with different disease combination characteristics. LCA breaks through the previous comorbidity assessment approach based solely on the number of diseases, helping to classify multiple coexisting chronic diseases into guided recommendations for chronic disease management based on expert interpretation. Secondly, the study was based on large-scale, nationally representative survey data of the middle-aged and elderly populations. Lastly, the study subdivided physical activity into moderate-intensity and high-intensity, and investigated its association with different coexistence patterns of multiple diseases, providing a basis for personalized and stratified intervention strategies and theoretical support for the comprehensive management of chronic diseases.

## 5 Conclusion

This study revealed associations between different levels of PA on different types of chronic disease multimorbidity patterns. The study suggests that moderate PA is associated with a lower risk of Multisystem Pattern and may be beneficial in the prevention and management of metabolic conditions. Although high-intensity PA may reduce the risk of metabolic and multisystem diseases, excessive PA may increase the risk for patients with high blood pressure, digestive disorders, and skeletal disorders. For middle -aged adults with chronic diseases in China, the intensity of PA should be reasonably adjusted according to their specific disease types and health conditions to maximize the health level and reduce the occurrence and progress of chronic diseases. These findings can inform future directions for effective interventions for managing chronic diseases, especially regarding the formulation of PA prescriptions, which requires further exploration of the appropriate intensity of PA under different disease patterns.

## Data Availability

Publicly available datasets were analyzed in this study. This data can be found here: the data for this study were extracted from CHARLS, a publicly available database provided by Peking University (https://charls.pku.edu.cn/).

## References

[1] Forman D Maurer M Boyd C Brindis R Salive M Horne F Multimorbidity in older adults with cardiovascular disease. *J Am Coll Cardiol.* (2018) 71:2149–61. 10.1016/j.jacc.2018.03.022 29747836 PMC6028235

[2] Tyack Z Frakes K Barnett A Cornwell P Kuys S McPhail S. Predictors of health-related quality of life in people with a complex chronic disease including multimorbidity: a longitudinal cohort study. *Qual Life Res.* (2016) 25:2579–92. 10.1007/s11136-016-1282-x 27048497

[3] Salisbury C Johnson L Purdy S Valderas J Montgomery A. Epidemiology and impact of multimorbidity in primary care: a retrospective cohort study. *Br J Gen Pract.* (2011) 61:e12–21. 10.3399/bjgp11X548929 21401985 PMC3020068

[4] Banatvala N Akselrod S Bovet P Mendis S. *The Who Global Action Plan for the Prevention and Control of Ncds 2013–2030. Noncommunicable Diseases.* Milton Park, MA: Routledge (2023). p. 234–9.

[5] Mair F Gallacher K. Multimorbidity: what next? *Br J Gen Pract.* (2017) 67:248–9. 10.3399/bjgp17X690965 28546391 PMC5442926

[6] Sherrington C Michaleff Z Fairhall N Paul S Tiedemann A Whitney J Exercise to prevent falls in older adults: an updated systematic review and meta-analysis. *Br J Sports Med.* (2017) 51:1750–8. 10.1136/bjsports-2016-096547 27707740

[7] Zhai T Goss J. Health system reform in china: the challenges of multimorbidity. *Lancet Glob Health.* (2020) 8:e750–1. 10.1016/S2214-109X(20)30225-4 32446340

[8] Bull F Al-Ansari S Biddle S Borodulin K Buman M Cardon G World health organization 2020 guidelines on physical activity and sedentary behaviour. *Br J Sports Med.* (2020) 54:1451–62. 10.1136/bjsports-2020-102955 33239350 PMC7719906

[9] Lee I Shiroma E Lobelo F Puska P Blair S Katzmarzyk P. Effect of physical inactivity on major non-communicable diseases worldwide: an analysis of burden of disease and life expectancy. *Lancet.* (2012) 380:219–29. 10.1016/S0140-6736(12)61031-9 22818936 PMC3645500

[10] Chudasama Y Khunti K Zaccardi F Rowlands A Yates T Gillies C Physical activity, multimorbidity, and life expectancy: a uk biobank longitudinal study. *BMC Med.* (2019) 17:108. 10.1186/s12916-019-1339-0 31186007 PMC6560907

[11] He L Biddle S Lee J Duolikun N Zhang L Wang Z The prevalence of multimorbidity and its association with physical activity and sleep duration in middle aged and elderly adults: a longitudinal analysis from china. *Int J Behav Nutr Phys Act.* (2021) 18:77. 10.1186/s12966-021-01150-7 34112206 PMC8194125

[12] Albuquerque A Borges-Silva F Da Silva Borges EG Pereira AP Dantas EHM. Physical activity: relationship to quality of life and memory in older people. *Sci Sports.* (2017) 32:259–65. 10.1016/j.scispo.2016.09.006

[13] Zeng Z Bian Y Cui Y Yang D Wang Y Yu C. Physical activity dimensions and its association with risk of diabetes in middle and older aged chinese people. *Int J Environ Res Public Health.* (2020) 17:7803. 10.3390/ijerph17217803 33113802 PMC7663282

[14] Li S Zhang J Yang Y. Correlation between the physical activity volume and cognitive and mental capacity among older adult people in china: a cross-sectional study based on the 2020 charls database. *Front Public Health.* (2024) 12:1462570. 10.3389/fpubh.2024.1462570 39635213 PMC11614726

[15] IPAQ. *Guidelines for Data Processing and Analysis of the International Physical Activity Questionnaire (IPAQ) - Short Form*. (2004). Available online at: https://www.physio-pedia.com/images/c/c7/Quidelines_for_interpreting_the_IPAQ.pdf (accessed September 18, 2025).

[16] Li X Zhang W Zhang W Tao K Ni W Wang K Level of physical activity among middle-aged and older chinese people: evidence from the china health and retirement longitudinal study. *BMC Public Health.* (2020) 20:1682. 10.1186/s12889-020-09671-9 33172439 PMC7653852

[17] Chen L Woo J Assantachai P Auyeung T Chou M Iijima K Asian working group for sarcopenia: 2019 consensus update on sarcopenia diagnosis and treatment. *J Am Med Dir Assoc.* (2020) 21:300–7. 10.1016/j.jamda.2019.12.012 32033882

[18] Gansevoort R Correa-Rotter R Hemmelgarn B Jafar T Heerspink H Mann J Chronic kidney disease and cardiovascular risk: epidemiology, mechanisms, and prevention. *Lancet.* (2013) 382:339–52. 10.1016/S0140-6736(13)60595-4 23727170

[19] Porter A Lash J Xie D Pan Q DeLuca J Kanthety R Predictors and outcomes of health–related quality of life in adults with ckd. *Clin J Am Soc Nephrol.* (2016) 11:1154–62. 10.2215/CJN.09990915 27246012 PMC4934840

[20] Xia J Wang L Ma Z Zhong L Wang Y Gao Y Cigarette smoking and chronic kidney disease in the general population: a systematic review and meta-analysis of prospective cohort studies. *Nephrol Dial Transplant.* (2017) 32:475–87. 10.1093/ndt/gfw452 28339863

[21] Yuan S Larsson S. Epidemiology of sarcopenia: prevalence, risk factors, and consequences. *Metabolism.* (2023) 144:155533. 10.1016/j.metabol.2023.155533 36907247

[22] Hu E Lazo M Rosenberg S Grams M Steffen L Coresh J Alcohol consumption and incident kidney disease: results from the atherosclerosis risk in communities study. *J Ren Nutr.* (2020) 30:22–30. 10.1053/j.jrn.2019.01.011 30850190 PMC6728235

[23] Lo C Lee P. Prevalence and impacts of poor sleep on quality of life and associated factors of good sleepers in a sample of older chinese adults. *Health Qual Life Outcomes.* (2012) 10:1–07. 10.1186/1477-7525-10-72 22709334 PMC3445836

[24] Huang Y Xia W Ge Y Hou J Tan L Xu W Sleep duration and risk of cardio-cerebrovascular disease: a dose-response meta-analysis of cohort studies comprising 3.8 million participants. *Front Cardiovasc Med.* (2022) 9:907990. 10.3389/fcvm.2022.907990 36237900 PMC9551171

[25] Da Silva A de Mello R Schaan C Fuchs F Redline S Fuchs S. Sleep duration and mortality in the elderly: a systematic review with meta-analysis. *Bmj Open.* (2016) 6:e8119. 10.1136/bmjopen-2015-008119 26888725 PMC4762152

[26] Petersen K Qualter P Humphrey N. The application of latent class analysis for investigating population child mental health: a systematic review. *Front Psychol.* (2019) 10:1214. 10.3389/fpsyg.2019.01214 31191405 PMC6548989

[27] Collins L Lanza S. *Latent Class and Latent Transition Analysis: With Applications in the Social, Behavioral, and Health Sciences.* Hoboken, NJ: John Wiley & Sons (2009).

[28] Berlin K Williams N Parra G. An introduction to latent variable mixture modeling (part 1): overview and cross-sectional latent class and latent profile analyses. *J Pediatr Psychol.* (2014) 39:174–87. 10.1093/jpepsy/jst084 24277769

[29] Miettunen J Nordström T Kaakinen M Ahmed A. Latent variable mixture modeling in psychiatric research – a review and application. *Psychol Med.* (2016) 46:457–67. 10.1017/S0033291715002305 26526221

[30] Xu L Xue C Yang K Chen L Chen X Xie X A latent class analysis of community-based rehabilitation needs among chinese older adults: a mixed study protocol. *Front Public Health.* (2024) 11:1301752. 10.3389/fpubh.2023.1301752 38283286 PMC10811259

[31] Wang M Deng Q Bi X Ye H Yang W. Performance of the entropy as an index of classification accuracy in latent profile analysis: a monte carlo simulation study. *Xin Li Xue Bao.* (2017) 49:1473–82. 10.3724/SP.J.1041.2017.01473 37113526

[32] Sen S Cohen A. An evaluation of fit indices used in model selection of dichotomous mixture irt models. *Educ Psychol Meas.* (2024) 84:481–509. 10.1177/00131644231180529 38756464 PMC11095322

[33] Wang M Chai P. Study on the chronic disease comorbidities pattern and the distribution of catastrophic health expenditure among the elderly in china. *Chinese Health Economics.* (2024) 43:47–50.

[34] Cerqueira É Marinho DA Neiva HP Lourenço O. Inflammatory effects of high and moderate intensity exercise—a systematic review. *Front Physiol.* (2020) 10:489354. 10.3389/fphys.2019.01550 31992987 PMC6962351

[35] Son W Park H Jeon B Ha M. Moderate intensity walking exercises reduce the body mass index and vascular inflammatory factors in postmenopausal women with obesity: a randomized controlled trial. *Sci Rep.* (2023) 13:20172. 10.1038/s41598-023-47403-2 37978254 PMC10656478

[36] Arem H Moore S Patel A Hartge P Berrington D Visvanathan K Leisure time physical activity and mortality: a detailed pooled analysis of the dose-response relationship. *JAMA Intern Med.* (2015) 175:959–67. 10.1001/jamainternmed.2015.0533 25844730 PMC4451435

[37] Perissiou M Borkoles E Kobayashi K Polman R. The effect of an 8 week prescribed exercise and low-carbohydrate diet on cardiorespiratory fitness, body composition and cardiometabolic risk factors in obese individuals: a randomised controlled trial. *Nutrients.* (2020) 12:482. 10.3390/nu12020482 32075010 PMC7071220

[38] Ahmadi M Clare P Katzmarzyk P Del P Lee I Stamatakis E. Vigorous physical activity, incident heart disease, and cancer: how little is enough? *Eur Heart J.* (2022) 43:4801–14. 10.1093/eurheartj/ehac572 36302460 PMC9726449

[39] Kokkinos P Faselis C Franklin B Lavie C Sidossis L Moore H Cardiorespiratory fitness, body mass index and heart failure incidence. *Eur J Heart Fail.* (2019) 21:436–44. 10.1002/ejhf.1433 30779281

[40] Franklin B Eijsvogels TM. A narrative review on exercise and cardiovascular disease: physical activity thresholds for optimizing health outcomes. *Heart and Mind.* (2023) 7:34–9. 10.4103/hm.hm_1_23

[41] Rader E Naimo M Ensey J Baker B. High-intensity stretch-shortening contraction training modifies responsivity of skeletal muscle in old male rats. *Exp Gerontol.* (2018) 104:118–26. 10.1016/j.exger.2018.02.009 29438735

[42] Kerkhoff A Moreira L Fuchs F Fuchs S. Association between hypertension and musculoskeletal complaints: a population-based study. *J Hypertens.* (2012) 30:2112–7. 10.1097/HJH.0b013e3283588268 22922700

[43] Coylewright M Reckelhoff J Ouyang P. Menopause and hypertension: an age-old debate. *Hypertension.* (2008) 51:952–9. 10.1161/HYPERTENSIONAHA.107.105742 18259027

[44] Zanchetti A Facchetti R Cesana G Modena M Pirrelli A Sega R. Menopause-related blood pressure increase and its relationship to age and body mass index: the simona epidemiological study. *J Hypertens.* (2005) 23:2269–76. 10.1097/01.hjh.0000194118.35098.43 16269969

[45] Guo X Zhao B Chen T Hao B Yang T Xu H. Multimorbidity in the elderly in china based on the china health and retirement longitudinal study. *PLoS One.* (2021) 16:e255908. 10.1371/journal.pone.0255908 34352011 PMC8341534

[46] Chen Y Chen Z Lu Y Zheng Y Wang W. Analysis of differences in smoking types among resident of quanzhou city in 2020. *Chinese J Health Educ.* (2025) 41:421–5. 10.16168/j.cnki.issn.10029982.2025.05.007

[47] Wang C Xiao D. 2020 report on health hazards of smoking in china: an updated summary. *Chin Circulation J.* (2021) 36:937–52. 10.3969/j.issn.1000-3614.2021.10.001

[48] Jeon H Salinas D Baker D. Non-linear education gradient across the nutrition transition: mothers’ overweight and the population education transition. *Public Health Nutr.* (2015) 18:3172–82. 10.1017/S1368980015001640 26054756 PMC4640944

[49] Smith W Anderson E Salinas D Horvatek R Baker DP. A meta-analysis of education effects on chronic disease: the causal dynamics of the population education transition curve. *Soc Sci Med.* (2015) 127:29–40. 10.1016/j.socscimed.2014.10.027 25459208

[50] Capistrant B Moon J Glymour M. Spousal caregiving and incident hypertension. *Am J Hypertens.* (2012) 25:437–43. 10.1038/ajh.2011.232 22189941 PMC3836043

[51] Liu L Xu N Wang L. Moderating role of self-efficacy on the associations of social support with depressive and anxiety symptoms in chinese patients with rheumatoid arthritis. *Neuropsychiatr Dis Treat.* (2017) 13:2141–50. 10.2147/NDT.S137233 28860771 PMC5558879

[52] Sharma R Dow-Fleisner S Struik L. Preventing and addressing youth vaping in british columbia, canada: evidence from triangulation of a scoping review of vaping policy and qualitative interviews with school-aged youth. *Prev Med Rep.* (2025) 51:102988. 10.1016/j.pmedr.2025.102988 39990200 PMC11847037

[53] Gelman A. Struggles with survey weighting and regression modeling. *Stat Sci.* (2007) 22:153–64. 10.1214/088342306000000691

[54] Winship C Radbill L. Sampling weights and regression analysis. *Sociol Methods Res.* (1994) 23:230–57. 10.1177/0049124194023002004

