# Supplementary material for: Association between physical activity and chronic disease multimorbidity patterns in Chinese middle-aged and older adults
Source: Front Med (Lausanne). 2025 Sep 29;12:1582846. doi: 10.3389/fmed.2025.1582846 (PMC12516536; doi:10.3389/fmed.2025.1582846)
Supplement: Supplementary file 1 [file Data_Sheet_1.pdf]

Table S1 Results of chronic multimorbidity by latent classes analysis

Diseases	Class1: Metabolic Pattern	Class2: Multisystem Pattern	Class3: Hypertension-Digestive-Musculoskeletal Pattern
Hypertension	0.802	0.541	0.236
Dyslipidemia	0.676	0.429	0.094
Diabetes	0.404	0.213	0.049
Cancer	0.028	0.044	0.02
Lung Cancer	0.038	0.621	0.065
Liver Disease	0.078	0.226	0.034
Heart Disease	0.401	0.524	0.071
Stroke	0.17	0.145	0.021
Kidney Disease	0.14	0.3	0.047
Digestive Disorders	0.316	0.634	0.241
Psychiatric Disorders	0.034	0.093	0.013
Memory Disorders	0.096	0.146	0.013
Arthritis	0.432	0.695	0.297
Asthma	0.004	0.356	0.010

Table S2 Definitions and Functions of Model Fit Indices in Latent Class Analysis

Abbreviation	Full Term	Definition & Function
AIC	Akaike Information Criterion	AIC is an information-theoretic model selection criterion. It evaluates model fit . Lower values indicate better model fit[1].
BIC	Bayesian Information Criterion	It imposes a stronger penalty on model complexity than AIC. Lower BIC suggests better fit[2].
aBIC	Sample-size Adjusted BIC	aBIC adjusts BIC for sample size, offering more accurate model evaluation in moderate-sized samples[3].
LMR	Lo–Mendell–Rubin Adjusted Likelihood Ratio Test	LMR compares a model with k classes to one with k-1 classes. It is mainly used to determine the optimal number of classes in latent class models (such as mixture IRT models), and is particularly suitable for comparisons between nested models[4].
BLRT	Bootstrap Likelihood Ratio Test	BLRT is a likelihood ratio test based on the bootstrap method, which evaluates the differences between models with adjacent numbers of classes by generating an empirical distribution through resampling. It addresses the failure of traditional likelihood ratio tests in mixture models due to non-standard distributions, providing a more reliable basis for determining the number of classes[4].

Explanation of Fitting Differences:

Fitting differences refer to the statistical improvement when moving from a model with k-1 classes to one with k classes. Both LMR and BLRT are designed to evaluate this. A significant p-value indicates better fit of the k-class model, thus it is preferred[3].

REFERENCES

1 Li W, Nyholt DR. Marker selection by akaike information criterion and bayesian information criterion. *Genet. Epidemiol.* 2001;21:S272-7.

2 Schwarz G. Estimating the dimension of a model. *The Annals of Statistics* 1978;6:461-4, 4.

3 Sclove SL. Application of model-selection criteria to some problems in multivariate analysis. *Psychometrika* 1987;52:333-43.

4 Sen S, Cohen AS. An evaluation of fit indices used in model selection of dichotomous mixture irt models. *Educ. Psychol. Meas.* 2024;84:481-509.

	Freq.	%
997 Don't Know	2,858	16.18
Total	17,662	100.00

da030: During Last Month Average Hours of Actual Sleep

Mean	SD	Min	Max	Obs
5.96	2.14	-1.00	24.00	19,355

da031: During Last Month Time for a Nap after Lunch

Mean	SD	Min	Max	Obs
42.20	45.73	-1.00	300.00	19,355

da032_1_: Intensive Physical Activity More than 10 Mins Each Time

	Freq.	%
1 Yes	6,886	35.58
2 No	12,467	64.42
Total	19,353	100.00

da032_2_: Moderate Physical Activity More than 10 Mins Each Time

	Freq.	%
1 Yes	10,759	55.59
2 No	8,594	44.41
Total	19,353	100.00

da032_3_: Light Physical Activity More than 10 Mins Each Time

	Freq.	%
1 Yes	14,879	76.89
2 No	4,472	23.11
Total	19,351	100.00

da033_1_: Days with Intensive Physical Activity

Mean	SD	Min	Max	Obs
4.83	2.17	1.00	7.00	6,886

da033_2_: Days with Moderate Physical Activity

Mean	SD	Min	Max	Obs
5.14	2.17	1.00	7.00	10,759

da033_3_: Days with Light Physical Activity

Mean	SD	Min	Max	Obs

5.94	1.75	1.00	7.00	14,879

da034_1_: Time with Intensive Physical Activity

	Freq.	%
1 <2 Hours	1,826	26.52
2 >=2 Hours	5,060	73.48
Total	6,886	100.00

da034_2_: Time with Moderate Physical Activity

	Freq.	%
1 <2 Hours	6,205	57.67
2 >=2 Hours	4,554	42.33
Total	10,759	100.00

da034_3_: Time with Light Physical Activity

	Freq.	%
1 <2 Hours	9,515	63.95
2 >=2 Hours	5,364	36.05
Total	14,879	100.00

da035_1_: Time with Intensive Physical Activity

	Freq.	%
1 <30 Minutes	332	18.18
2 >=30 Minutes	1,494	81.82
Total	1,826	100.00

da035_2_: Time with Moderate Physical Activity

	Freq.	%
1 <30 Minutes	1,582	25.50
2 >=30 Minutes	4,623	74.50
Total	6,205	100.00

da035_3_: Time with Light Physical Activity

	Freq.	%
1 <30 Minutes	1,814	19.06
2 >=30 Minutes	7,701	80.94
Total	9,515	100.00

da036_1_: Time with Intensive Physical Activity

	Freq.	%
1 <4 Hours	1,775	35.08
2 >=4 Hours	3,285	64.92

	Freq.	%
Total	5,060	100.00

da036_2_: Time with Moderate Physical Activity

	Freq.	%
1 <4 Hours	2,540	55.79
2 >=4 Hours	2,013	44.21
Total	4,553	100.00

da036_3_: Time with Light Physical Activity

	Freq.	%
1 <4 Hours	3,376	62.94
2 >=4 Hours	1,988	37.06
Total	5,364	100.00

da037_1_: Purpose for Intensive Physical Activity

	Freq.	%
1 Job Demands	5,032	73.08
2 Entertainment	165	2.40
3 Exercise	1,153	16.74
4 Others, Please Specify	536	7.78
Total	6,886	100.00

da037_2_: Purpose for Moderate Physical Activity

	Freq.	%
1 Job Demands	4,867	45.24
2 Entertainment	377	3.50
3 Exercise	2,408	22.38
4 Others, Please Specify	3,107	28.88
Total	10,759	100.00

da037_3_: Purpose for Light Physical Activity

	Freq.	%
1 Job Demands	4,420	29.71
2 Entertainment	1,588	10.67
3 Exercise	7,476	50.25
4 Others, Please Specify	1,395	9.38
Total	14,879	100.00

da038_s1: Activities in Last Month

	Freq.	%
0 No	13,165	68.03
1 Interacted With Friends	6,186	31.97
Total	19,351	100.00

da038_s2: Activities in Last Month

	Freq.	%
0 No	16,517	85.35
2 Played Ma-jong, Played Chess, Played Cards, or Went to Community Club	2,834	14.65
Total	19,351	100.00

da038_s3: Activities in Last Month

	Freq.	%
0 No	16,362	84.55
3 Provided Help to Family, Friends, or Neighbors Who Do Not Live With You	2,989	15.45
Total	19,351	100.00

da038_s4: Activities in Last Month

	Freq.	%
0 No	18,023	93.14
4 Went to a Sport, Social, or Other Kind of Club	1,328	6.86
Total	19,351	100.00

da038_s5: Activities in Last Month

	Freq.	%
0 No	18,891	97.62
5 Took Part in a Community-Related Organization	460	2.38
Total	19,351	100.00

da038_s6: Activities in Last Month

	Freq.	%
0 No	18,743	96.86
6 Done Voluntary or Charity work, or Cared for a Sick or Disabled Adult Who Does Not Live With You	608	3.14
Total	19,351	100.00

da038_s7: Activities in Last Month

	Freq.	%
0 No	19,044	98.41
7 Attended an Educational or Training Course	307	1.59
Total	19,351	100.00

da038_s8: Activities in Last Month

	Freq.	%
0 No	18,988	98.12
8 Others, Please Specify	363	1.88

Total	19,351	100.00

da038_s9: Activities in Last Month

	Freq.	%
0 No	9,296	48.04
9 None of These	10,055	51.96
Total	19,351	100.00

da039_1_: Frequency of Activities[1]

	Freq.	%
1 Almost Daily	1,900	30.71
2 Almost Every Week	1,348	21.79
3 Not Regularly	2,938	47.49
Total	6,186	100.00

da039_2_: Frequency of Activities[2]

	Freq.	%
1 Almost Daily	715	25.23
2 Almost Every Week	829	29.25
3 Not Regularly	1,290	45.52
Total	2,834	100.00

da039_3_: Frequency of Activities[3]

	Freq.	%
1 Almost Daily	216	7.23
2 Almost Every Week	522	17.46
3 Not Regularly	2,251	75.31
Total	2,989	100.00

da039_4_: Frequency of Activities[4]

	Freq.	%
1 Almost Daily	782	58.89
2 Almost Every Week	242	18.22
3 Not Regularly	304	22.89
Total	1,328	100.00

da039_5_: Frequency of Activities[5]

	Freq.	%
1 Almost Daily	30	6.52
2 Almost Every Week	105	22.83
3 Not Regularly	325	70.65
Total	460	100.00
